# Surgical Treatment of Proximal Segmental Occlusion of the Internal Carotid Artery

**DOI:** 10.1155/2019/2976091

**Published:** 2019-01-02

**Authors:** Srdjan Babić, Slobodan Tanasković, Mihailo Nešković, Predrag Gajin, Dragoslav Nenezić, Predrag Stevanović, Nikola Aleksić, Milorad Ševković, Nenad Ilijevski, Predrag Matić, Petar Popov, Goran Vučurević, Dragana Unić-Stojanović, Djordje Radak

**Affiliations:** ^1^Department of Vascular Surgery, Institute for Cardiovascular Disease “Dedinje”, Belgrade, Serbia; ^2^Faculty of Medicine, University of Belgrade, Belgrade, Serbia; ^3^Department of Surgery, Clinical Center Dr Dragisa Misovic, Belgrade, Serbia

## Abstract

**Purpose:**

To present the feasibility, safety, and efficacy of carotid endarterectomy in patients with type II internal carotid artery occlusions, including the long-term outcomes.

**Methods:**

From March 2008 to August 2015, 74 consecutive patients (48 men with a mean age of 65.1 ± 8.06 years) underwent carotid endarterectomy because of internal carotid artery (ICA) segmental occlusions. These were verified with preoperative carotid duplex scans (CDS) and CT angiography (CTA). Also, brain CT scanning was performed in all these patients. The indication for treatment was made jointly by a vascular surgeon, neurologist, and an interventional radiologist in a multidisciplinary team (MDT) context. After successful treatment, all the patients were followed-up at 1, 3, 6, and 12 months, then every 6 months thereafter.

**Results:**

The most common symptom at presentation was transient ischaemic attack (TIA) in 49 patients (66.2%), followed by stroke in the past six months in the 17 remaining patients (23%). Revascularisation of the ICA with endarterectomy techniques was performed successfully in all the patients with an average clamp time of 11.9 min. All the procedures were performed under general anaesthesia in combination with a superficial cervical block. The early complication rate was 8.1% and included two cardiac events (2.7%) (one rhythm disorder and one acute coronary syndrome), three TIAs (4.1%), and one intracerebral hemorrhage (1.3%). Only one patient with the intracerebral hemorrhage died 5 days after surgery giving a postoperative mortality of 1.3% for this series. During the follow-up period (mean 50.4 ± 31.3 months), the primary patency rates at 1, 3, 5, and 7 years were 98.4%, 94.9%, 92.9%, and 82.9%, respectively. Likewise, the survival rates were 98.7%, 96.8%, 89%, and 77.6%, respectively. Ultrasound Doppler controls during follow-up detected 8 ICA restenoses; however, only 3 of these patients required further endovascular treatment.

**Conclusions:**

Carotid endarterectomy of internal carotid artery (ICA) segmental occlusion is a safe and effective procedure associated with acceptable risk and good long-term results. Therefore, the current guidelines which do not recommend carotid endarterectomy in this patient group should be reassessed, with the requirement for ongoing large-scale randomized controlled trials to compare CEA with best medical therapy in this patient cohort.

## 1. Introduction

Almost twenty-five years ago, several large randomized studies showed that the surgical treatment of high-grade internal carotid artery (ICA) stenoses can be successful in the prevention of cerebrovascular events [1, 2]. In the past, the general body of opinion was that the case of an occluded carotid artery bared no significant risk of ipsilateral stroke for the patient, and surgical treatment was therefore believed to be excessive and unnecessary [3]. However, several studies have shown that the risk of an acute neurologic event in patients with an occluded ICA is still substantially high [4, 5]. One can also argue that these patients are being exposed to prolonged hypoperfusion and potentially have an increased risk of developing vascular dementia [6]. There is also a considerable risk of distal embolization from the ICA stump, and 4–18% of patients may develop a syndrome of chronic ocular ischaemia [7]. Finally, ICA occlusion can manifest as ongoing syncopal episodes [8].

Not all patterns of ICA occlusion are the same, as they differ in angiographic and intraoperative findings. Kniemeyer et al. classified extracranial ICA occlusions into three types: type I as subtotal stenosis or pseudoocclusion; type II as a total occlusion of proximal internal carotid artery, with delayed interrogates flow in its cervical portion and carotid siphon, through unusual collateral vessels; and type III as complete extracranial ICA occlusion [9]. The earliest studies of surgical therapy in treating symptomatic ICA occlusions showed disappointing results with fatal complications, mainly that of postoperative intracranial hemorrhage [10, 11]. Later on, a prospective randomized trial analyzed the feasibility of extracranial–intracranial bypass procedures but showed no benefit in ipsilateral stroke prevention compared with medical therapy alone [12, 13]. Endovascular treatment showed favorable results, but there is ongoing concern about the disruption of the atherothrombotic plaque during the initial crossing of the lesion, with a consequential risk of embolic stroke [14]. On the other hand, some authors concluded that thromboendarterectomy of the occluded ICA can be a safe and effective procedure in preventing stroke with adequate patient selection [9, 15, 16].

Although established guidelines do not currently recommend CEA in this specific patient group, one has to acknowledge that the evidence does suggest that there are increased neurological risks for patients with surgically untreated ICA occlusions and also that surgical treatment has demonstrated positive results in this group. We are a highly specialized, busy, and experienced carotid surgery unit, with excellent results and a demonstrable safe track record. Therefore, we felt obligated duty to further investigate if CEA can be safe and beneficial in the long term for such patients. We believe that only by such ambitious research and the stretching of established boundaries can major progress in evidence-based carotid interventions be achieved.

This current study was therefore undertaken to review our 8-year experience of surgical treatment in patients with short segmental occlusion of proximal extracranial ICA and to evaluate its safety, short-term patency and long-term patency, clinical success rates, and any predictive risk factors.

## 2. Methods

### 2.1. Patients

From March 2008 to August 2015, carotid endarterectomy (CEA) was performed in 5,516 patients at the University Cardiovascular Clinic in Serbia. Among these, we identified 74 patients (48 men with a mean age of 65.1 ± 8.1 years) with extracranial ICA occlusions. Data were collected prospectively as a part of our hospital's ongoing vascular database. Patients were all admitted for investigation and treatment of their cerebrovascular disease, based upon their previous CDS findings.

**Figure 1:**
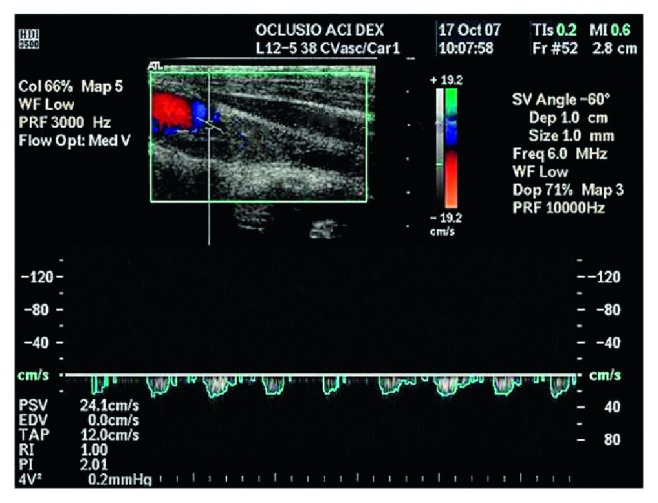
CDS findings of the ICA occlusion.

**Figure 2:**
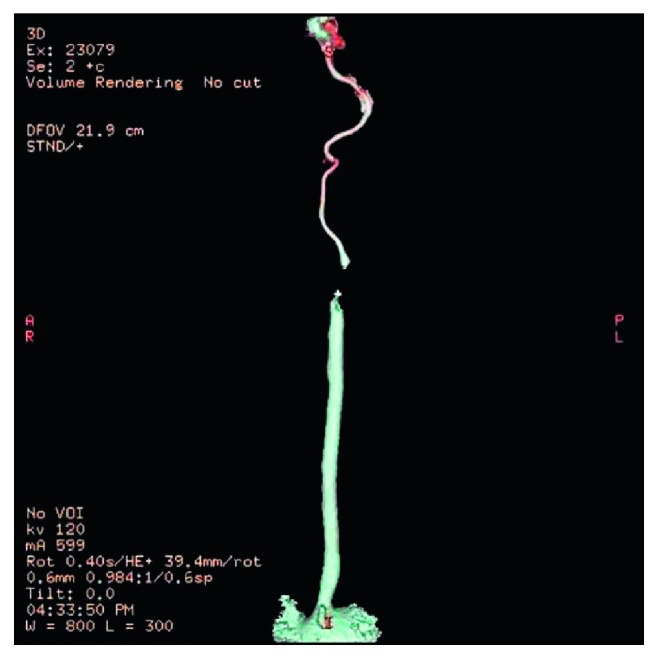
MDCT angiography of type II segmental ICA occlusion.

**Figure 3:**
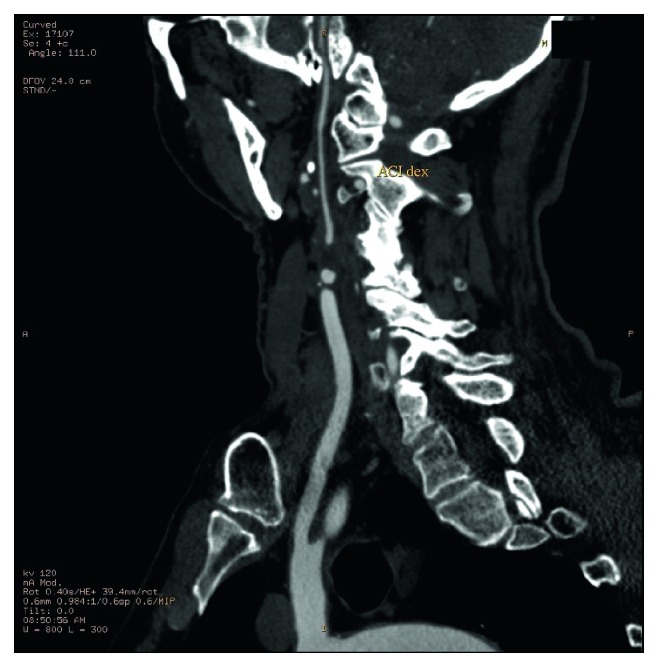
MDCT angiography of type II segmental ICA occlusion (multiplanar reconstruction).

On admission, all patients were subjected to a full physical examination and a CDS of the extracranial carotid, vertebral, and subclavian arteries (Figure 1). Subsequently, we performed multidetector computerised tomographic (MDCT) angiography (Lightspeed VCT; GE Healthcare, Milwaukee, WI, USA) of the supra-aortic branches and intracranial vessels, along with brain computerised tomography (CT) scans in all the patients with the finding of an ICA occlusion. Type II segmental occlusion of extracranial ICA (Figures 2 and 3), defined as a total proximal occlusion of ICA with fully patent cervical and intracranial parts, was described in every patient [9]. All the decisions regarding surgical treatment were made by a multidisciplinary team consisting of a vascular surgeon, neurologist, and an interventional radiologist.

The contralateral ICA status for each patient was graded as follows: mild stenosis (<50%) in 37 patients and moderate stenosis (50%–69%) in eight patients. There were no patients with severe contralateral ICA stenoses (70%–99%) in the study group. In 29 (40%) patients, previous carotid stenting (*n*=7) or carotid endarterectomy (*n*=22) had been performed on the contralateral ICA. The neurological examination was performed before and after the carotid endarterectomy by two experienced neurologists.

### 2.2. Symptoms

There were 49 patients with previous transient ischaemic attacks (TIA), who had the following manifestations: amaurosis fugax in 19 (25.8%), contralateral paresis in 11 (14.9%), speech disturbance in 10 (13.5%), diplopia in 3 (4%), syncope in 3 (4%), and headache in 3 (4%). We defined TIA as a brief episode of neurologic dysfunction caused by focal brain ischaemia, without the imaging evidence of infarction. Seventeen patients (23%) had prior stroke in the past 6 months (four recurrent strokes), 4 (5.4%) patients had global neurological symptom, and 4 patients (5.4%) were asymptomatic. In addition, 8 (10.7%) patients with a segmental ICA occlusion and a high-grade vertebral artery stenosis had complained of occasional vertigo.

All patients gave informed consent for use of their data in the production of this paper. The study was approved by our local ethical committee.

### 2.3. Surgical Treatment and Administration of Drugs

Surgery was performed under general anaesthesia in combination with a superficial cervical block. During the procedure, the patients were systemically anticoagulated (heparin in doses of 100 units/kg, with the activated clotting time (ACT) kept between 250 and 300 seconds). Eversion endarterectomy of ICA was performed in all patients, with a mean clamping time of 11.9 min. All procedures were performed without the use of shunting, given the preknown ICA occlusion. Our modified eversion endarterectomy technique that implies an ellipsoid ICA transection with prolonged incision on the medial ICA wall provides a successful eversion endarterectomy for longer lesions under direct vision to prevent residual plaque or dissection. Also, during preoperative planning, we precisely evaluate the plaque length in the ICA with the MDCT angiography. According this diagnostic tools, average lesion length in our study group was 28.4 ± 6.2 mm.

All patients received mannitol and/or hypertonic saline solution intra- and postoperatively to prevent brain edema and the blood pressure BP was medically maintained between its normal preoperative value and 20% above in all cases. Patients were extubated and neurologically examined immediately after the procedure whilst still in the operating room. Upon admission, in every consecutive patient, acetylsalicylic acid (100 mg/d) and high dose of statins (40 mg/d) were administrated. After surgery, acetylsalicylic acid (100 mg/d) was continued, whereas ticlopidine (250 mg twice daily) or clopidogrel (75 mg/d) were given on the third postoperative day. Patients experiencing side effects, such as bleeding, appetite loss, headache, or palpitation, were treated with a single agent. Other cardiovascular medications were used when clinically indicated. Statins were administered in 72 of 74 patients (97.2%) on discharge.

Detailed demographic data are shown in Table 1.

### 2.4. Follow-Up and Definitions

During follow-up, patients were examined by the attending surgeon, and the duplex ultrasound controls were performed at 1, 3, and 6 months in the first year and every 6 months thereafter (or whenever new neurological symptoms appeared). Technical success was defined as a blood flow reconstitution after ICA revascularisation with eversion CEA. Clinical success was defined as technical success related to periprocedural events from the initiation of the procedure through the first 24-hour postoperative period and with symptom resolution beyond 24 hours after the procedure. Ischaemic cerebrovascular events (strokes, TIA), intracerebral hemorrhage, and worsening of the symptoms were assessed.

### 2.5. Statistical Analysis

Standard descriptive statistics were used. Kaplan–Meier curves were constructed to assess mortality and stenosis-free survival during the follow-up period. Cox univariate and multivariate analyses were performed to assess the predictors of ICA restenosis and survival during the follow-up period. Individual differences were considered to be statistically significant for *P* < 0.05. SPSS version 18.0 (SPSS Inc, Chicago, Ill) was used for all statistical calculations.

## 3. Results

Technical success with eversion CEA was achieved in all of the patients, with a mean clamping time of 11.9 min. In seven patients (9.5%), initial percutaneous transluminal angioplasty (PTA) of a high-grade vertebral artery stenosis was performed to enable adequate cerebral perfusion, while CEA was then performed in the next step. After CEA, complete resolution of neurological symptoms was noted in 44 of 49 patients who had preoperative focal neurological disorders. In the remaining 5 patients, there was complete symptom resolution over the next three days.

Neurological complications occurred in 4 (5.4%) patients. Postprocedural TIA was noted in two patients (one diplopia and one hemiparesis). Both patients were fully recovered, and postoperative CT brain was unremarkable. No specific therapy was therefore given. In another two patients (2.7%) with a postoperative neurological event (≤24 h after the surgery), acute ICA thrombosis was confirmed, and they required an immediate return to the theatre. In both patients, we performed Dacron tubular graft interposition (6 mm) between CCA and ICA. The first patient fully recovered following this. The primary indication for surgery in second one was a stroke in progression, and CT brain after our reintervention showed intracerebral hemorrhage and unfortunately the patient died 5 days after surgery.

Two patients had cardiac events in the early postoperative period (one rhythm disorder and one acute coronary syndrome), giving a total early complication rate of 8.1%. After the initial 30-day period, there were no other neurological or wound complications.

### 3.1. Follow-Up Data

The median follow-up period was 50.4 ± 31.3 months (range, 2–127 months). Three patients (4.1%) were lost during the follow-up period. Ultrasound Doppler controls during follow-up detected eight (10.6%) ICA restenoses: 5 mild (≤50%), 1 moderate (50–69%), and 2 severe (≥70). Three patients required endovascular treatment because of these symptomatic moderate and severe restenoses after 20, 42, and 66 months (after the carotid endarterectomy). The patients who received endovascular treatment had recurrent symptoms of vertigo and instability following therapy. Another five patients had asymptomatic mild restenoses. They were treated by best medical therapy and simply followed-up every 3 months using CDS.

**Figure 4:**
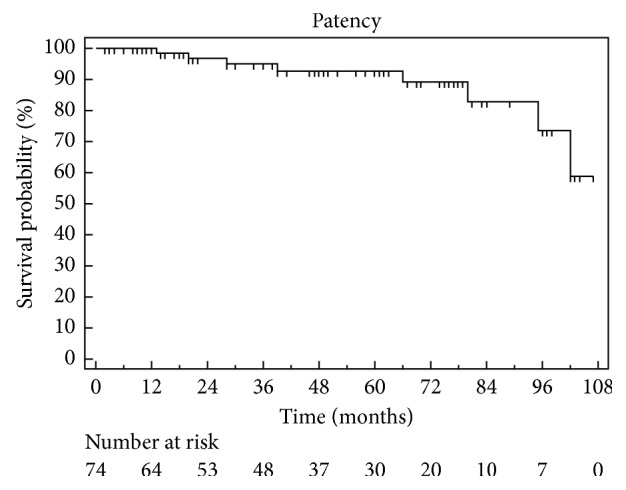
Kaplan–Meier patency rate curve.

**Figure 5:**
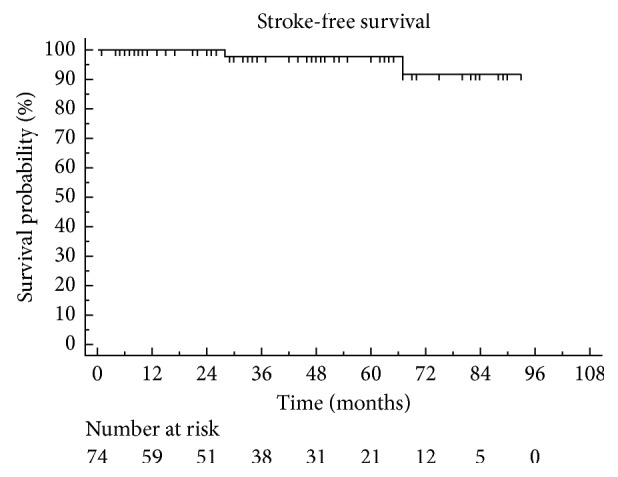
Kaplan–Meier stroke-free survival rate.

Figures 4 and 5 show Kaplan–Meier curves of restenosis and stroke-free survival after successful CEA, respectively. Univariate analysis evaluating the following factors failed to identify any variable predictors of successful carotid revascularisation: age; sex; risk factors for vascular disease; presence of subclavian, vertebral, cardiac, and peripheral artery disease; length; and side of the occlusion and clamp time.

**Figure 6:**
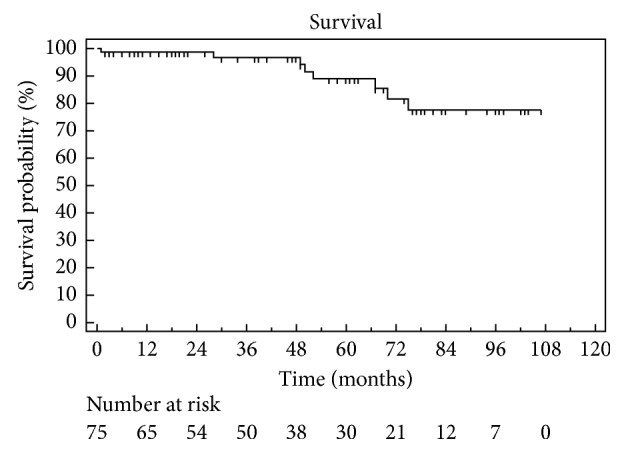
Kaplan–Meier survival rate.

Eight patients (10.6%) died during follow-up: 2 from fatal carotid territory stroke of uncertain cause, 4 because of myocardial infarction, and 2 because of malignancy as can be seen from Kaplan–Meier survival curve (Figure 6).

## 4. Discussion

The benefits of carotid endarterectomy in patients with high-grade ICA stenosis was established through large randomized controlled trials, both in symptomatic and asymptomatic patients [1, 2, 17]. Although the management we describe in this paper is not according to current guidelines [18], considering our vast experience [19] (over 15000 carotid endarterectomies performed in last 15 years), we wanted to stretch the boundaries of carotid surgery for cerebrovascular disease treatment and see if we could improve future practice. According to this current study, our approach optimizes brain perfusion and also reduces the risk of recurrent strokes in the territory of the occluded ICA. Furthermore, we aimed to delay/eliminate the onset of vascular dementia in such patients by salvaging the occluded carotid artery and improving global cerebral perfusion.

Our current study shows the results of our 8-year experience of carotid endarterectomy for ICA occlusion. The main finding of our study is that patients with segmental ICA occlusions have low periprocedural risk and indeed good long-term results. Patency rates after at 1, 3, 5, and 7 years were 98.4%, 94.9%, 92.9%, and 82.9%, respectively. The stroke-free survival after 1, 3, and 5 years was 100%, 97%, and 97%, respectively. During follow-up (mean, 50.4 ± 31.3 months; range, 2–127 months), only eight patients developed restenosis, of which only 3 required further endovascular treatment. The univariate analysis failed to identify any variable predictive factor associated with restenosis after carotid artery unblocking.

In general, ICA occlusion is often treated with medical therapy alone, despite the presence of ipsilateral hemispheric symptoms, and only a small number of studies offer good results with more invasive treatment methods [10, 11, 15, 16]. At first, the risk of ipsilateral acute neurologic event was attributed only to hypoperfusion of the brain tissue distal to the occluded ICA. Gomensoro et al. postulated that after complete thrombosis of the artery there is no possibility for emboli to pass into distal circulation [3]. However, later on several more theories were presented that explained the frequent neurologic symptomatology in patients with an occluded ICA, such as distal thrombus propagation into the cerebral arteries, embolization from occluded carotid stumps and even embolization from contralateral carotid arteries through collateral circulation [4, 16, 20]. In a recent paper, the natural history of ICA occlusion was analyzed in 153 patients diagnosed by CDS [20]. After the mean follow-up period of 35 months (IQR 14–61 months), 29% of the patients had TIA or stroke and 25% in the territory of the occluded ICA. On the other hand, they described a 10.3% (8 of 77 patients) recanalization rate in patients that underwent repeated scans at a median of 53 months, pointing out the potential significance of this phenomenon in medical treatment of carotid artery disease. To this day, there have been only a few papers concerning endovascular recanalization of chronically occluded ICA [14, 21]. The procedures consisted of using equipment for recanalization of chronic coronary artery occlusions, with both pre- and postdilation and embolic protection devices when permitted by the vessel diameter. Studies included a small number of patients (30 and 54), but with strict criteria of segmental ICA occlusions confirmed by selective angiography. Technical success, defined as crossed and stented lesion with a final residual diameter stenosis <20% and TIMI distal antegrade flow grade 3, was achieved in 65–73% of cases. While there was only one ipsilateral minor stroke in both series, one study [21] presented 3 cases of late major complications. In addition, the in-stent reocclusion rate ranged from 5.7–6.2%, although all of the patients were asymptomatic. Future RCTs are therefore necessary to establish the effectiveness and safety of endovascular treatment of patients with segmental ICA occlusions.

The benefit of thromboendarterectomy in patients with acute neurological symptomatology and a suspected recent ICA occlusion was noted in several papers. In a retrospective study, 29 patients with ICA occlusion were operated on within 8 days of symptom onset [15]. The ligation of ICA had to be performed in 5 of 29 (17%) patients due to chronic occlusion or absence of backflow. In one patient (3.4%) there was a conversion into hemorrhagic stroke, and this was the only perioperative death. Also the authors of this study attributed the improved patency rates (79%) in the follow-up period to better patient selection, compared to their former study (46%). They suggested that proper identification should lean towards the patients with mild or improving neurological deficits and patients with distally patent ICAs. Another paper compared the results of different surgical treatments of symptomatic ICA occlusions in 90 patients, within 14 days of symptom presentation [16]. In group 1, patients were submitted to both internal and external carotid endarterectomy, while in group 2, only external carotid artery endarterectomy was performed due to the inability to improve the ICA lumen patency. In 7 out of 90 patients, adjunctive procedures had to be done to improve inflow to the carotid bifurcation. In patients with stroke there was improvement in 64% of the cases in group 1, while there was none in group 2 and calculated 5-year stroke-free rate was 71% in group 1 and 60% in group 2.

Kniemeyer et al. classified extracranial ICA occlusions into three different types based on angiographic and intraoperative findings and evaluated the results of its surgical treatment in 128 patients [9]. Type I was termed pseudoocclusion and was defined as a subtotal stenosis, while type II and III were termed segmental occlusions. Type II was defined as a short proximal ICA occlusion with the majority of its cervical part visible, and type III was defined as an occlusion of the whole cervical part of the artery with a visible petrous part. The overall perioperative stroke rate was 7.8% and mortality rate was 2.3%. The overall ipsilateral TIA and stroke rate were 5.6% and 3.4%, respectively, at a mean follow-up of 41.1 ± 29.91 months, whilst the overall annual TIA and stroke rate were 1.8% and 0.9%, respectively. The authors concluded that despite the relatively high perioperative complication rates, surgical treatment should be taken into consideration in patients with extracranially occluded ICA due to the significant reduction in acute neurological event rate after treatment. In the previously mentioned study [9], patients were submitted to special protocol of cerebral angiography with delayed sequencing technique to differentiate between the occlusion types.

Conventional angiogram is an invasive procedure which can show only one side of an occluded blood vessel in a single projection. It is on very rare occasions that a distal part can be shown in a single session, if the collateral blood flow exists, while with MDCT all vessels are shown in a single session. In the case of type II ICA occlusion, the part of the vessel distal to the occlusion is shown retrogradely, which cannot be seen using conventional angiography. Considering that the patency of the ICA distal to the occluded segment determines the course of treatment, MDCT angiography has shown multiple beneficial purposes in day-to-day practice. The obvious question in these patients is whether their ICA occlusions were truly occluded or whether “trickle” flow had not been identified either because of interpretation error or technological difficulties. Thanks to its high resolution, the presence and absence of contrast can confidently determine if there is occlusion. Indeed, we strongly assert that the patients in our study did indeed have definite carotid occlusions and that they were not very tight carotid stensoses that were misdiagnosed radiologically. Indeed, out of all diagnostic tools, including conventional angiography, MDCT has the highest sensitivity in diagnosing pseudoocclusions and even the string sign. A study comparing CDS, MDCT, and MRA with DSA concluded that MDCT has excellent diagnostic accuracy with sensitivity, specificity, positive, and negative predictive values up to 100% [22]. MDCT constitutes a valuable modality for the evaluation of carotid disease, with accurate grading of stenosis and fewer complications compared to DSA. MDCT's ability to readily detect stenosis severity and plaque quality is attributed to the availability of specialized three-dimensional reformatting software such as multiplanar reconstruction (MPR), maximum intensity projection (MIP), and volume rendering (VR), with the overall accuracy of MDCTA, with all techniques deployed, showing 93.9% sensitivity and 98.7% specificity, similar to DSA [22]. With improvements in temporal and spatial resolution, MDCT can complete a wide range of scanning in a short period of time and, compared to MRI, is less affected by motion artifacts owing to its shorter acquisition time [23]. MDCT also allows for multiplanar reconstructions in the axial, sagittal, and coronal planes, as well as high spatial and contrast resolution which allows for high, submillimeter spatial resolution [24] and precise differentiation between high-grade stenosis and actual segmental occlusion. The 64-section CTA imaging protocol for carotid arteries yields high-quality studies in 95% of cases [25]. As a result, modern noninvasive modalities such as MDCT are gradually replacing DSA. In our study, in order to minimize the risk of misdiagnosing the ICA occlusions, all scans were performed either by a single vascular radiologist or by a vascular sonographer. Another important finding is that all patients in our series had a fully patent distal part of ICA without lesion and a fully patent and functional circle of Willis. This finding encouraged us to start with brain revascularisation in the case of segmental ICA occlusion.

By exploring the natural history of ICA occlusion in 167 patients, Faught et al. concluded that there was a significant incidence of subsequent stroke, which was strongly related to the degree of stenosis of the contralateral ICA [26]. Although there were no patients in our study with high-grade contralateral ICA stenoses, 29 out of 74 patients were already treated for contralateral carotid disease, either by stenting or endarterectomy. From our perspective, the treatment of contralateral symptomatic high-grade ICA stenoses still seems more beneficial compared to the treatment of occluded ICA.

Our study has shown that high patency rates are attainable even after a five- or seven-year period, despite the prior severe atherosclerotic changes in the carotid arteries. However, our opinion is that revasculariation of the segmentally occluded ICA should be reserved for more specialized centers with extensive experience in carotid surgery [27].

### 4.1. Study Limitation

The main study limitation is the obvious lack of a definitive control group. During the same period, we detected another 9 patients with segmental ICA occlusions whom were treated with best medical therapy alone (BMT). After fully detailed cardiac and neurological examination, indication for BMT was made because of their extensive medical comorbidities which made them extremely poor surgical candidates. This specific group of patients had a very heavy burden of disease: six already had a disabling stroke (modified Rankin score > 3), and two had significant cardiac comorbidity. In addition, one other patient refused surgery anyway. We did not consider attempting EVT in these patients because: (1) the high reported complication rates in recent studies and (2) the presence of disabling stroke in the area of the occluded artery. Indeed, during the study period, 7 of these comorbid patients had a fatal stroke (after 4, 5, 7, 8, 9, 14, and 18 months). Another two patients died within two years due to cardiac failure. This high-mortality rate in patients with ICA occlusion treated with BMT is shown in previous studies [4, 5]. However, because of the small number of patients treated with BMT alone, we could not perform comparative analysis (surgery vs best medical therapy).

## 5. Conclusion

The surgical treatment of patients with segmental extracranial ICA occlusion is a safe and effective operation, with acceptable perioperative complication rates and a high postoperative survival rate. In the future, more attention should be paid to the investigation and management of segmental ICA occlusion, since it is surgically treatable and BMT is not the only available option. There are two important prerequisites for successful segmental ICA occlusion CEA: (1) visualization of distal ICA and (2) fully patent and functional circle of Willis. Currently, this cohort of patients is typically being managed by best medical therapy alone, and this paper suggests that surgery may potentially be a better option for selected patients. Similarly, perhaps it is time for the guidelines to be reassessed with regards to how best this patient cohort should be managed. Of course, there is still a need for much larger prospective randomized controlled trials on the subject, but this paper should stimulate further interest in this fascinating and rapidly developing area of vascular surgery.

## Figures and Tables

**Table 1:** Demographic data of the patients.

Variable	*n*=74	%
Smoking	60	81.08
Arterial hypertension	66	89.19
Hyperlipoproteinemia	64	86.49
Diabetes mellitus	18	24.32
Family history	43	58.11
Coronary artery disease	27	36.49
Peripheral artery disease	26	35.13

## Data Availability

The data used to support the findings of this study are available from the corresponding author upon request.
